# The Effect and Mechanism of External Use Ulcer Powder on Diabetic Mice

**DOI:** 10.7759/cureus.26903

**Published:** 2022-07-15

**Authors:** Bingyang Wang, Wenlin Xie, Xiaofang Lu, Jian Song, Hongsen Liang

**Affiliations:** 1 Department of Pathology, Seventh Affiliated Hospital, Sun Yat-sen University, Shenzhen, CHN; 2 Department of Thoracic Surgery, Seventh Affiliated Hospital, Sun Yat-sen University, Shenzhen, CHN

**Keywords:** mice, wyk, effect, diabetes foot ulcer, ulcer

## Abstract

Objective

Through the preparation of the diabetic mice skin ulcer model, we investigated the effect of Mongolian medicine external ulcer powder (WYK) on the treatment of diabetic skin ulcers and the expression of angiogenesis-related factors such as vascular endothelial growth factor (VEGF) and extracellular regulated protein kinases (ERK).

Methods

Thirty male clean Kunming mice were randomly divided into normal control group (group C), diabetic control group (group HC), and diabetic topical ulcer powder group (group HW). After successful modeling in the HC group and the HW group, the rats in the HW group were given external ulcer powder, which was applied to the back of the mice once a day. In addition, the rats in group C and group HC were treated with gentamicin injection external application once a day. The mice were sacrificed on the 3rd, the sixth, and the ninth day of dosing, and samples were taken. The adopted methods included protein immunoblotting (western blot) and reverse transcription-polymerase chain reaction (RT-PCR). The expression differences of angiogenesis-related factors such as VEGF and ERK in the repair process were detected. SPSS 13 software was used to analyze the results of angiogenesis-related factors VEGF and ERK.

Results

Comparison of VEGF and ERK Contents

The serum VEGF content of mice in the HC group was significantly lower than that in the C group on days 3, 6, and 9 (p <0.05). The VEGF content in the HW group was significantly higher than that in the HC group (p <0.05). The content of ERK in serum was basically consistent with that of VEGF. The results of the western blot assay were consistent with those of the RT-PCR assay.

Conclusion

WYK can effectively promote the healing of skin ulcer wounds in diabetic mice, accelerate the proliferation of granulation tissue, enrich the contents of capillary blood tubes and collagen fibers, and increase the microvascular content. WYK can improve the expression level of VEGF and ERK in the serum of mice and advance the peak value of protein expression.

## Introduction

The rapid increase in the prevalence of diabetes in the world has led to an increase in the incidence of diabetes-related complications. In 2021, an estimated 466 million adults worldwide had diabetes, and the forecast shows that this number will rise to 552 million by 2030 [1-3]. Foot disease has high morbidity and mortality, which is the most common cause of hospitalization in diabetic patients. The lifetime risk of a diabetic foot ulcer is between 15% and 25%, and the annual incidence rate is about 2% [4-6]. The risk of lower limb amputation in a person with diabetes is estimated to be 23 times that of a person without diabetes [7-10]. These slow and refractory wounds bring substantial medical expenses to patients and also significantly affect the quality of life of diabetic patients. So far, the pathogenesis of diabetic foot ulcers such as neuropathy and lower limb is still being explored.

A chronic diabetic wound is a problematic point in clinical treatment. One of its main pathological mechanisms is the local microvascular formation and dysfunction in the wound, and the expression level of endogenous growth factors such as vascular endothelial growth factor (VEGF) and extracellular regulated protein kinases (ERK) decreases [11-15]. Therefore, promoting the expression of growth factors in wound tissues plays an essential role in wound healing. In the complex and highly coordinated process of wound vascularization, the regulatory role of growth factors plays a key role. For a long time, the theory of Mongolian medicine and effective prescription has been applied to treat skin ulcers, and good clinical effects have been achieved [16,17]. However, Mongolian medicine, especially compound medicine, has complex active ingredients, and many unknown areas remain unknown. Therefore, it is urgent to carry out more high-quality clinical trials and animal experiments for further exploration. Mongolian medicine external ulcer powder (WYK), formerly known as Qiwei Kuchuang powder, also known as Hatagoqi-7 and Gamujuer, is a commonly used clinical prescription of Mongolian medicine. In 1971, Inner Mongolia Ancient Medical Institute compiled it into the Mongolian version of the Mongolian Medical Examination Party. In 1984, it was formally incorporated into Inner Mongolia Mongolian Medicine Standards by the Health Department of Inner Mongolia Autonomous Region [18-21]. WYK is one of the most commonly used clinical prescriptions of Mongolian medicine. It is a heritage of ethnic medicine at a reasonable price. This medicine is convenient, safe, and economical to use, and there are fewer chances of relapse after being cured. Therefore, it is being popularized and used continuously in the clinic. However, there is no related study regarding the use of WYK for diabetic foot ulcers. In this experiment, the Mongolian medicine, WYK, is used for the first time in the experimental study of diabetic mice foot ulcers to observe the healing and involution of mouse skin ulcers and the expression of endogenous growth factors such as VEGF and ERK.

## Materials and methods

Experimental animal 

A total of 30 Kunming mice, specific-pathogen-free (SPF) grade, male, weighing 28.5±3.50 g, aged 6-8 weeks, were purchased from Sibei Fu (Beijing, China) Experimental Animals Technology Co., Ltd with the license number: SCXK (Beijing) 2011-0004. The name of the local animal ethics committee or NIH guidelines statement is Inner Mongolia Medical University Scientific Research and Experimental Animal Ethics Committee (IMMU-IACUC-2020 -B1089). The mice were fed in a laminar flow rack (SPF grade, no specific pathogen grade), and the temperature was controlled at 22±2℃. The relative humidity was 40%-60%.

Drug

WYK (production enterprise: Inner Mongolia Mongolian Medicine Co., Ltd., z15020458), ingredients: ice stone, realgar, cinnabar, Yinzhu, shidiming, ice sheet, artificial musk), gentamycin.

Drugs used were streptozotocin (STZ) (Sigma Company, USA), sodium citrate (Beijing Solebao Company), citric acid (Beijing Solebao Company), chloral hydrate (Beijing Solebao Company), polyclonal antibody against VEGF (Fuzhou Maixin Company), high-efficiency RIPA tissue lysate (Beijing Solebao Company), BCA protein quantitative kit (Jiangsu Biyuntian Biotechnology Research Institute), SDS-PAGE gel preparation kit (Jiangsu Biyuntian Biotechnology Research Institute), protein pre-dyeing marker (Thermo Company, USA), 4× protein loading buffer (Beijing Solebao Company), ERK monoclonal antibody (ABCAM Company, USA), DNA marker (Beijing Solebao Company), and PCR primer (Shanghai Shenggong Company).

Drugs such as PVDF membrane (Millipore), 15 ml centrifuge tube (Corning Company, USA), 1.8ml frozen storage tube (Corning Company, USA), clean animal feeding cabinet (Suzhou and Hangzhou live animal testing equipment factory), HF32-U86-86℃ ultra-low temperature refrigerator (Heal Force Company, Hong Kong), Sigma-4K15 desktop centrifuge (SIG-MA Company, USA), ZWY-211b constant temperature shaker (Shanghai Zhicheng Company), ES-215 high-pressure steam sterilizer (Tomy Company, Japan), blood glucose meter (Johnson & Johnson Medical Equipment Co., Ltd.), animal tissue total RNA extraction kit (Beijing Tiangen Biochemical Technology Co., Ltd.), One-step RT-PCR kit (Beijing Tiangen Biochemical Technology Co., Ltd.), DYY-8C electrophoresis apparatus (Beijing Liuyi Instrument Factory), vertical electrophoresis tank and electron transport tank (Beijing Liuyi Instrument Factory), GBOX HR gel image analysis system (Gene Company Limited), and Quantum One-4.0.3 image analysis (Bio-RAD), were also used.

Experimental methods

Animal Grouping and Model Preparation Method 

The experimental animals were divided into three groups randomly: the control group (Group C, n = 10) and the diabetes group (HD group, n = 20). The diabetic group was randomly divided into two groups: the control group (HC group, n = 10) and the diabetic external ulcer powder group (HW group, n = 10). Group C was fed with common feed for 30 days, and the HD group was fed with high fat and sugar feed for 30 days. After fasting for 12 hours, the mice in group C were weighed and injected with sodium citrate buffer in the abdominal cavity. Stz70mg/kg was injected into the abdominal cavity of HD mice (formula: take citrate A solution and sodium citrate B solution according to the proportion of 1:1.32, prepare buffer solution, dissolve STZ, prepare 1% solution, avoid light, prepare with an ice bath, and inject it in 15 mins). Blood glucose is measured by blood collection from the tail vein after three days. Blood glucose ≥16.7 mmol/l was regarded as successful modeling. Then blood glucose was measured weekly, and a stable diabetic mice model was selected. The mice were injected intraperitoneally with 1% pentobarbital sodium (0.05mg/g). After anesthesia, the hair of the back of the mice was shaved with a razor. Under the sterile operation environment, the area of the back of the mice was 8×. The skin defect of 8 mm was recorded on day 0. In the experiment, each group was kept in a cage, five in each cage and 10 in each group. Mice in each group were fed freely and cleaned regularly.

Administration and sampling 

The administration and materials were given through gentamicin injection in the HW group. External ulcer powder was applied to the ulcer of the back of mice once a day, and the dosage was 10 mg/cm2. In groups C and HC, gentamycin injection was given as external treatment, 0.9% sodium chloride injection was used to smear the ulcer site once a day, and the dosage was 0.1 ml/cm2.

Observation of ulcer healing area 

Photos of the skin ulcer surface of mice were taken with Image-Pro Plus V6.0 to obtain the ulcer area on the 3rd, 6th, and 9th day after administration and treating it. Ulcer healing of each group was then compared.

The expression of VEGF mRNA and ERK mRNA was detected by RT-PCR.

The total RNA of experimental animal tissues was extracted using the experimental steps of the Trizol method (Beijing Tiangen Biochemical Technology Co., Ltd.). Real-time fluorescence quantitative RT-PCR was carried out according to the operation steps of one-step RT-PCR. The sample loading system was 10×RT-PCR Buffer 5 μl, Ntdx2 ultrapure ul, 5×RT-PCR enhancer 10ul, RNasin (40 U/μl) 0.5 u, HotMaster Taq DNA Polymerase (2.5 U/μl) 2.5 ul, Quant Rtase (for one-step) 0.5 μl, upstream specific primer (10 μm) 3 μl, downstream specific primer (10 μm) 3 μl, RNA template 1 μG total RNA, RNase-free water, makeup water to 50 μl and total system 50 μl.

RT-PCR reaction steps: reverse transcription temperature at 500 ℃ for 30 mins, PCR initial denaturation at 940 ℃ for 2 mins, denaturation at 940 ℃ for 0.5 mins, annealing at 50-600 ℃ for 0.5 mins, and extension at 650 ℃ for 1.5 mins. A total of 35 cycles were performed from steps 3-5. The final extension temperature was 650 ℃ for 10 mins, and the insulation temperature was 40 ℃. Agarose gel (concentration 1.2%) electrophoresis was performed on PCR products and was detected under a gel imaging system.

Detection of intracellular ERK and VEGF by western blot

Take the ulcer surface tissue and add tissue lysate (50 mmol / L Tris-HCl, pH＝7.5, 150 mmol/L NaCl, 1 mmol/L EDTA, 1% NP-40, 0.25% sodium deoxycholate, 1 mmol/L PMSF, 0.1% SDS), then extract the protein, and conduct quantitative loading of BCA protein 20 μg. A total of 120V gel (12%) electrophoresis, membrane transfer at 4 ℃ for 2 h, sealing for 2 h, TBST washing membrane for 10 mins, adding rabbit anti-mouse VEGF (1:1000) antibody, incubating overnight at 4 ℃, TBST washing membrane 3 × 10 mins, sheep anti-rabbit IgG was incubated at 37 ℃ (1:300) for 1.5 h, ECL was developed, Kodak film was exposed for 10 s, and the results were scanned into the computer and processed by image analysis software to obtain the relative absorbance value. 1.3 statistical processing all statistical processing is completed in SPSS13.0 software.

Analysis

All statistical analysis was done using SPSS 13.0 software. The data are expressed by (X ± S), and the analysis of variance of repeated measurement design is used to study the data. LSD test is used for the square difference pairwise comparison. Inspection level α＝ 0.05, with p <0.05 as the judgment standard with statistical significance.

## Results

General observation of mice 

The mice in the HD group were observed to lose weight after successful model making, have dry hair, no luster, and have poor mental state. The volume for water intake, eating state, and urine quantity increased. The body weight of group C mice was not significantly changed; the mental state was as good as before, the hair was smooth and bright, and the water intake, eating state, and urine quantity were not significantly changed.

Wound healing rate 

On the 3rd, 6th, and 9th day of administration, the wound healing rate of mice in group H was significantly lower than that in group C (P <0.05). On the 3rd and 6th days, the wound healing rate of mice in the HW group was significantly lower than that in the C group (P <0.05). On the 9th day, the wound healing rate of mice in the HW group was lower than that in the C group, but the difference was not statistically significant (P >0.05). The wound healing rate of mice in the HW group was higher than that of group H on the 3rd day of dosing, but the difference was not statistically significant (P >0.05). On the 6th and 9th days, the wound healing rate of mice in the HW group was significantly higher than that of group H (Table 1).

**Table 1:** Changes in wound healing rate in mice at different time points in each group (X±S, %). Note: * indicates there is a difference in wound healthing rate in mice from group C,  P <0.05; Δ indicates that wound healing rate is different from HW group and HC group, P < 0.05.

Group	N	Day 3	Day 6	Day 9
Group C	10	24.30±1.61	49.32±1.62	86.55±2.89
Group HC	10	19.32±1.31^*^	34.56±1.24^*^	49.67±1.26^*^
Group HW	10	22.14±1.12^*^	45.20±1.68^*^^Δ^	86.34±2.98^Δ^

The expression of ERK and VEGF was detected by western blot

ERK changes

**Figure 1:**
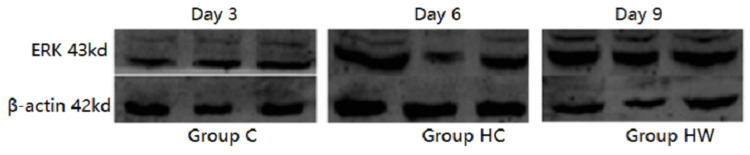
The results of western blot imaging of ERK protein in granulation tissue of mice wound on the 3rd, 6th, and 9th days of administration were obtained. ERK: Extracellular regulated protein kinases.

The grey value of ERK protein expression in the HC group was lower than that in the C group at each time point, and the difference was statistically significant (P <0.05). The grey value of HW histone expression was higher than that of the H group, and the difference was statistically significant on the 6th and 9th days (P < 0.05), which indicated that WYK could promote the expression of ERK. The grey value of HW histone expression was lower than that of group C, and the 3rd and 6th day difference had statistical significance (P < 0.05) (Table 2 and Figure 1).

**Table 2:** Western blot was used to detect the grey value of ERK/β-actin protein expression (X±S, kda). Note: * indicates there is a difference in wound healthing rate in mice from group C,  P <0.05; Δ indicates that wound healing rate is different from HW group and HC group, P < 0.05. ERK: Extracellular regulated protein kinases.

Group	N	Day 3	Day 6	Day 9
Group C	10	2.52±0.31	3.31±0.24	5.01±0.23
Group HC	10	1.70±0.30^*^	2.67±0.20^*^	2.34±0.18^*^
Group HW	10	2.32±0.28^*^	2.93±0.25^*^^Δ^	4.01±0.21^Δ^

VEGF changes 

**Figure 2:**
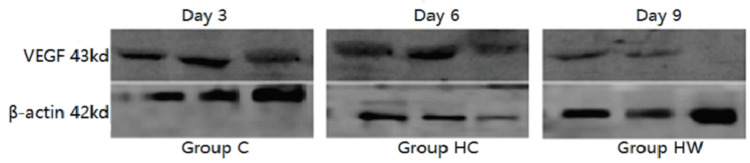
The results of western blot imaging of VEGF protein in granulation tissue of mice wound on the 3rd, 6th, and 9th days of administration were obtained. VEGF: Vascular endothelial growth factor.

The grey value of VEGF protein expression in group H was lower than that in group C at each time point, and the difference had a systematic significance (P <0.05). The grey value of HW histone expression was higher than that of the H group, and the difference was statistically significant on the 6th and 9th days (P <0.05), which indicated that WYK could promote the expression of VEGF. The grey value of egg white in the HW group was lower than that in the C group, and the difference was statistically significant on the 3rd day (P <0.05) (Table 3, Figure 2).

**Table 3:** Changes of VEGF in wound tissues of mice in each group at each time point (X±S, kda). Note: * indicates there is a difference in wound healthing rate in mice from group C,  P <0.05; Δ indicates that wound healing rate is different from HW group and HC group, P < 0.05. VEGF: Vascular endothelial growth factor.

Group	N	Day 3	Day 6	Day 9
Group C	10	0.78±0.06	1.45±0.07	1.56±0.01
Group HC	10	0.74±0.04^*^	0.87±0.04^*^	1.21±0.03^*^
Group HW	10	0.76±0.06^*^	1.28±0.08^*^^Δ^	1.47±0.02^Δ^

RT-PCR results

**Figure 3:**
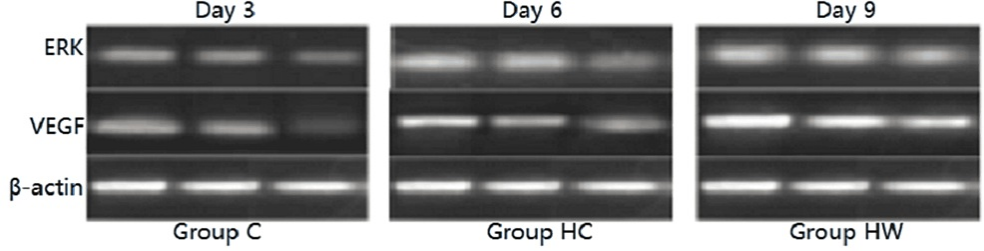
ERK and VEGF mRNA detected by RT-PCR. ERK: Extracellular regulated protein kinases; VEGF: Vascular endothelial growth factor; RT-PCR: Reverse transcription-polymerase chain reaction.

The grey value of ERK mRNA at each time point in group H was lower than that in group C, and the difference was statistically significant on the 3rd, 6th, and 9th days (P <0.05). The grey value of the HW group was higher than that of the H group, and the difference was statistically significant on the 6th and 9th days (P <0.05). The grey value of the HW group was lower than that of the C group on the 3rd and 6th days, and the difference was statistically significant (P <0.05) (Table 4 and Figure 3).

**Table 4:** Changes of ERK in wound tissues of mice in each group at each time point (X±S, bp). Note: * indicates there is a difference in wound healthing rate in mice from group C,  P <0.05; Δ indicates that wound healing rate is different from HW group and HC group, P < 0.05. ERK: Extracellular regulated protein kinases.

Group	N	Day 3	Day 6	Day 9
Group C	10	0.53±0.04	0.61±0.07	0.79±0.01
Group HC	10	0.46±0.03^*^	0.56±0.02^*^	0.61±0.03^*^
Group HW	10	0.50±0.02^*^	0.60±0.06^*^^Δ^	0.75±0.02^Δ^

The grey value of VEGF mRNA at each time point in group H was lower than that in group C, and the difference was statistically significant on the 3rd, 6th, and 9th days (P <0.05). The grey value of the HW group was higher than that of the H group, and the difference was statistically significant on the 6th and 9th days (P < 0.05). The grey value of the HW group was lower than that of the C group on the 3rd day, and the difference was statistically significant (P < 0.05) (Table 5).

**Table 5:** Changes of VEGF in wound tissues of mice in each group at each time point (X±S, bp). Note: * indicates there is a difference in wound healthing rate in mice from group C,  P <0.05; Δ indicates that wound healing rate is different from HW group and HC group, P < 0.05. VEGF: Vascular endothelial growth factor.

Group	N	Day 3	Day 6	Day 9
Group C	10	0.87±0.04	1.30±0.04	1.43±0.01
Group HC	10	0.73±0.05^*^	0.87±0.02^*^	1.14±0.03^*^
Group HW	10	0.76±0.08^*^	1.42±0.06^*^^Δ^	1.51±0.01^Δ^

## Discussion

The occurrence of a diabetic foot ulcer is the pathological result of many risk factors such as vascular disease, neuropathy, local infection, and plantar biomechanical changes. In the state of sustained high glucose, the dysfunction of cells and tissues, the injury of microvessels and peripheral blood vessels, and the decrease of neurotrophic factor expression are closely related to the occurrence of diabetic foot ulcers. At present, it has been found that the difficulty of wound healing is mainly related to the abnormal function of growth factors (FGFR, VEGF, etc.) that regulate neuroangiogenesis.

VEGF is an active peptide secreted by macrophages to promote the proliferation of vascular endothelial cells. It acts on vascular endothelial cells through paracrine function, promotes the synthesis of plasminogen activator and collagenase in endothelial cells, and participates in the proliferation of vascular endothelial cells. At present, it is the strongest angiogenic factor found, and it is involved in inducing angiogenesis, endothelial cell growth, and other important physiological and pathological processes. ERK is the first mitogen-activated protein kinase (MAPK), which is involved in regulating physiological and pathological processes such as cell proliferation, differentiation, and apoptosis. Studies have shown that the activated ERK1/2 signaling pathway can regulate the expression level of phosphorylated ERK1/2 and ATF2, and make ATF2 (the downstream substrate of ERK1/2 activating p38 signaling pathway) interact with each other to repair chronic refractory wounds jointly. RAS-MAPK is an important way of information transmission, and it plays a crucial role in gene expression regulation and cytoplasmic function. In eukaryotic cells, four MAPK signal transduction pathways have been identified, and one of them, the ERK signal transduction pathway, is the earliest and most complete classical pathway. It can be activated by various pathogenic factors, transducing signals from cell membrane surface receptors to nuclei and participating in a series of immune responses mediated by G-protein-coupled receptors. Therefore, the mechanism of FGFR's effect on the ERK signal transduction pathway in diabetic ulcers is studied to achieve active prevention and treatment of early diabetic ulcers. A high sugar environment can inhibit receptor phosphorylation and reduce the binding ability of growth factor receptors. This channel is inhibited in diabetic mice, making the wound difficult to heal. The above factors related to angiogenesis synergistically promote wound angiogenesis, thus promoting wound granulation tissue formation. Mongolian Medicine Ulcer Powder for External Use, formerly known as Qiwei Kuchuang Powder, also known as Gamujuer, is a commonly used clinical prescription of Mongolian medicine. It consists of seven kinds of medicines, namely mirabilite (ⅵ), cinnabar, concha Haliotidis, realgar, vermilion, borneol, and musk. It is a powder for external use, pink in color, bitter in flavor, and has the effects of diminishing inflammation and relieving pain, clearing away heat and toxic materials, promoting blood circulation, decreasing swelling, improving local blood circulation, promoting granulation tissue proliferation, and accelerating wound healing. In addition, its rich calcium ions participate in coagulation reaction and promote hemostasis of wound surface. It can also increase the tolerance of tissues to hypoxia and increase the body's immunity. It can also avoid local irritation of other drugs and avoid damaging the surrounding skin and other adverse reactions. It has a certain protective effect on the skin mucosa.

Although there is evidence that targeted interventions for diabetic foot ulcers and multidisciplinary care can reduce the loss of limbs, the progress has been slow so far [22-24]. Although the elderly population with glycosuria amputation is declining, the number of patients with type 2 diabetes has increased in some countries [25,26]. In this experiment, the diabetic mouse foot ulcer model was established to observe the curative effect of external ulcer powder on mouse foot ulcers and the expression of angiogenesis-related factors ERK and VEGF. The results showed that topical ulcer powder could significantly promote the healing of diabetic foot ulcers, but it could not reach the level of the control group. After using WYK, the expression levels of ERK and VEGF proteins, as well as ERK mRNA and VEGF mRNA in tissues, are significantly increased, but the increase is still less than that in the control group. Local ERK and VEGF in the diabetic control group were significantly lacking. Through the determination of ERK and VEGF, it can be determined that there is insufficient VEGF secretion in diabetic foot ulcers. Local use of WYK can promote the healing of diabetic foot ulcers and increase the expression levels of local ERK and VEGF.

Limitations

Due to this experimental model being a mouse, the mouse's weight is quite different from that of diabetic patients, so it is impossible to accurately estimate the exact dose of drugs applied to the human body, which is also the research direction of the follow-up work of this experiment. Follow-up experiments will focus on applying WYK in clinical practice and observe the actual effect and accurate dose of WYK in patients with a diabetic foot ulcer.

## Conclusions

WYK can improve the wound healing rate of diabetic skin ulcer mice, make the new granulation tissue grow well, and promote the proliferation of capillaries and collagen fiber production in granulation tissue. It also increases the content of microvessels in the wound and promotes wound healing by promoting angiogenesis in granulation tissue.

Using WYK can effectively increase the expression of VEGF and ERK in diabetic mice. ERK coordinates the normal proliferation of endothelial cells by regulating the cell cycle. It promotes VEGF capillaries to fuse into larger blood vessels. The above factors related to angiogenesis synergistically promote wound angiogenesis, granulation tissue formation, and wound healing.

## Appendices

**Table 6:** The English abbreviations and their meanings.

Abbreviations	Meaning
HE	Hematoxylin and eosin stain
WB	Western blot
IOD	Integral optical density
STZ	Streptozotocin
VEGF	Vascular endothelial growth factor
ERK	Extracellular regulated protein kinases
WYK	Mongolian medicine external ulcer powder
RNA	Ribonucleic acid
PCR	Polymerase chain reaction
